# Study protocol of a multicenter, randomized, non-inferiority, open-label study investigating the efficacy of a fixed-dose combination regimen of dapagliflozin/metformin versus co-administered dual therapy based on glycemic control, satisfaction and adherence in patients with newly diagnosed type 2 diabetes mellitus

**DOI:** 10.3389/fendo.2025.1690339

**Published:** 2025-11-04

**Authors:** Jiaxuan Wang, Ying Wei, Ping Shi, Ji Chen, Lingling Xu, Yinghong Kong, Jun Ye, Xueqin Wang, Juan Xu, Guang Wang

**Affiliations:** ^1^ Department of Endocrinology, Beijing Chaoyang Hospital of Capital Medical University, Beijing, China; ^2^ Department of Endocrinology, The People’s Hospital of Dingzhou, Dingzhou, China; ^3^ Department of Endocrinology, The People’s Hospital of Zhuji, Zhuji, China; ^4^ Department of Endocrinology, Shenzhen Hospital, Southern Medical University, Shenzhen, China; ^5^ Department of Endocrinology, The Second People’s Hospital of Changshu, Changshu, China; ^6^ Department of Endocrinology, The Second People’s Hospital of Hefei, Hefei, China; ^7^ Department of Endocrinology, The First People’s Hospital of Nantong, Nantong, China; ^8^ Department of Endocrinology, The People’s Hospital of Jiangyin, Jiangyin, China

**Keywords:** T2DM, newly diagnosed, dapagliflozin/metformin, FDC, co-administered dual therapy

## Abstract

**Introduction:**

The combination of dapagliflozin and metformin is commonly used as an initial therapy for patients with type 2 diabetes mellitus (T2DM) with high glycated hemoglobin (HbA1c) levels. Although bioequivalence has been established for the fixed-dose combination (FDC) of dapagliflozin/metformin extended-release (XR) compared to the co-administration of dapagliflozin and metformin XR, it remains uncertain whether the efficacy of dapagliflozin/metformin FDC is comparable to that of co-administration. Additionally, it is still unclear whether fixed-dose combinations offer advantages in terms of patient adherence and satisfaction. This study aims to compare the dapagliflozin/metformin XR FDC with co-administration of dapagliflozin and metformin XR for efficacy in terms of glycemic control, patient satisfaction, quality of life and adherence in Chinese patients with newly diagnosed T2DM.

**Methods and analysis:**

This multicenter, randomized, non-inferiority, open-label clinical trial enrolled 632 patients with T2DM (HbA1c 7.5–10%) in 35 research centers in China. After enrollment, the patients will be randomly assigned to receive either FDC treatment (10 mg dapagliflozin and 1000 mg metformin XR) or co-administered 10 mg dapagliflozin and 1000 mg metformin XR tablets for 24 weeks. The primary endpoint was the change in HbA1c level from baseline after 24 weeks of treatment. Secondary endpoints included the proportion of patients achieving HbA1c below 7.0%, absolute changes in fasting plasma glucose and postprandial glucose from baseline to week 24, the difference in patient satisfaction measured with the diabetes treatment satisfaction questionnaire, medication usage measured with adherence to refills and medications scale for diabetes between the two groups at week 24, change from baseline in diabetes quality of life questionnaire score at week 12 and week 24, and safety. Continuous glucose monitoring will also be used to evaluate the benefits of FDC compared with co-administration on glycemic variability.

**Discussion:**

This study, as the first of its kind, will provide comparative data on the efficacy of the dapagliflozin/metformin XR FDC and co-administration of dapagliflozin and metformin XR in terms of glycemic control, patient satisfaction, quality of life and adherence. These data will help clinicians make more informed decisions for patients with type 2 diabetes and may improve medication burden, treatment adherence, and satisfaction.

## 1 Introduction

The global prevalence of diabetes poses a significant threat to public health, with China having the highest number of patients with diabetes worldwide. From 2015 to 2017, the prevalence of diabetes among adults in China was 12.8% (1). Long-term blood glucose control is fundamental for diabetes management, and it is important to focus on cardiovascular and renal protection during the treatment process (2).

Metformin has firmly established itself as a cornerstone in the treatment of type 2 diabetes mellitus (T2DM) and remains the first-line medication in most clinical guidelines (3). It effectively lowers blood glucose by reducing hepatic glucose output and improving peripheral insulin resistance. It has benefits such as weight reduction, reduced risk of cardiovascular diseases associated with T2DM, synergistic effects in combination therapy, a good safety profile, and is cost-effective and widely available (3, 4). Sodium-glucose cotransporter 2 (SGLT2) inhibitors, a class of oral antidiabetic agents that have gained widespread application and have been consistently recommended by guidelines in recent years, include dapagliflozin, empagliflozin, canagliflozin, and ertugliflozin (5). These agents lower blood glucose levels by inhibiting SGLT2 in the proximal renal tubules, blocking glucose reabsorption, and promoting urinary glucose excretion (6). In addition to their glucose-lowering effects, SGLT2 inhibitors confer important non-glycemic benefits including renal hemodynamic regulation, decreased urinary protein excretion, blood pressure reduction, and weight loss (7). These non-glycemic pathways have a positive effect on patient treatment, with multiple large-scale clinical trials demonstrating the efficacy of SGLT2 inhibitors in reducing the risk of major adverse cardiovascular events, cardiovascular death, myocardial infarction, heart failure hospitalization, and all-cause mortality, as well as improving renal outcomes in T2DM patients with cardiovascular disease or at high cardiovascular risk (8–11). In addition, SGLT2 inhibitors have been shown to reduce cardiorenal and cardiovascular risks in patients with chronic kidney disease or heart failure, regardless of the presence or absence of diabetes (12, 13).

Glycemic control in patients with diabetes typically requires combination therapies. Studies have shown that initial combination therapy could achieve faster glycemic control and maintain its effects for a longer duration than sequential add‐on therapy (14, 15). The American Diabetes Association (ADA) 2025 Standards of Medical Care in Diabetes and Guidelines for the Prevention and Treatment of Diabetes Mellitus in China (2024 edition) recommended considering initial combination therapy, including fixed-dose combination (FDC), for patients whose glycated hemoglobin (HbA1c) is over 1.5% above the target level (2, 16). Furthermore, combination therapy was recommended for T2DM patients with the HbA1c ≥7.5%, according to the American Association of Clinical Endocrinology (AACE) Consensus Statement (2023) and Metabolic Disease Management Guideline for National Metabolic Management Center (MMC) (17, 18). Nowadays, low adherence to diabetes medication remains an ongoing problem (19). Studies have shown that medication adherence is inversely related to the number of medications (20), and compared to using separate drugs, fixed-dose combination therapy may significantly improve patient adherence to treatment (21). XIGDUO^®^ XR tablet is a FDC of dapagliflozin and metformin hydrochloride XR tablets, and studies have shown bioequivalence between FDC and co-administered dapagliflozin and metformin XR tablets (22). However, it remains uncertain whether the efficacy of dapagliflozin/metformin FDC is comparable to that of co-administration.

Currently, studies comparing FDC to co-administration are generally rare, and there are no head-to-head comparative studies between dapagliflozin/metformin FDC and the co-administration of dapagliflozin and metformin hydrochloride. Additionally, there is no direct evidence of the glycemic control efficacy of the FDC of dapagliflozin 10 mg and metformin XR 1000 mg in patients with T2DM, which is the only available dosage for dapagliflozin/metformin XR FDC in clinical use in China. Therefore, we designed a multicenter, randomized, non-inferiority, open-label clinical trial to compare the effects of dapagliflozin/metformin XR FDC with those of co-administration of dapagliflozin and metformin XR on glycemic control, patient satisfaction, quality of life, adherence, and safety. This study is the first of its kind and has the potential to provide crucial evidence supporting the use of FDCs, including dapagliflozin/metformin XR FDC. Additionally, it may offer valuable insights for clinical practice regarding the selection of treatment regimens for diabetes and improvement of patient adherence.

## 2 Methods and analysis

### 2.1 Study design

This was a 24-week, multicenter, randomized, non-inferiority, open-label clinical trial designed to compare the FDC regimen of dapagliflozin/metformin XR with dapagliflozin administered alongside metformin XR, based on glycemic control, patient satisfaction, and adherence in Chinese patients with T2DM. The study was conducted at 35 sites in China.

**Figure 1:**
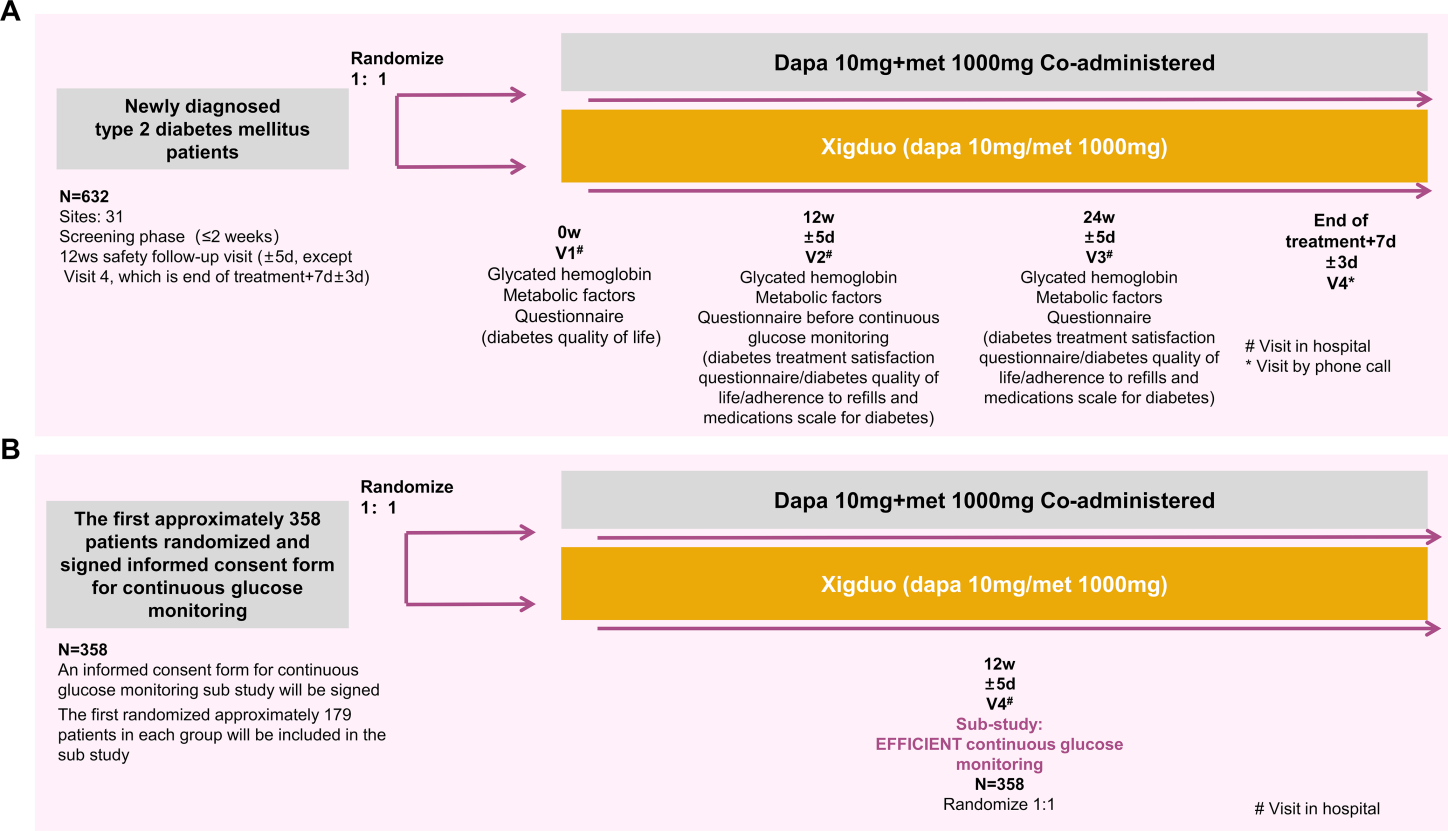
Study flowcharts for the main study **(A)** and the exploratory sub-study **(B)**.

The flowchart of the study is shown in **Figure 1**. After obtaining written informed consent during the screening period, the patients will be assessed for eligibility according to all applicable inclusion and exclusion criteria, and laboratory samples will be collected and submitted. Once the investigator confirms the patient’s eligibility, an HbA1c blood sample will be collected and tested by the central laboratory, with results available within two weeks. Eligible patients will undergo re-evaluation of their inclusion and exclusion criteria based on the test results. Eligible patients will be randomly assigned 1:1 to one of two treatment groups. From week 0, patients in the FDC group will receive treatment with an FDC regimen consisting of 10 mg dapagliflozin and 1000 mg metformin XR tablets for 24 weeks, while the co-administration group will receive 10 mg dapagliflozin tablets and 1000 mg metformin XR tablets for 24 weeks. Among the 632 randomly assigned subjects, the first approximately 179 subjects per group who signed the continuous glucose monitoring (CGM) informed consent will be included in the CGM sub-study, totaling approximately 358 patients. After completing all scheduled tests, examinations, and questionnaires at week 12, participants in the sub-study will undergo CGM (for approximately five days). Follow-up visits will be conducted every 12 weeks (± 5 days) during the treatment period, with an additional follow-up visit at 1 week (± 3 days) after the end of the treatment to evaluate efficacy and safety.

### 2.2 Participants

To be eligible for the study, participants must be 18 years of age or older at the time of signing the informed consent form and must be capable of signing the written informed consent voluntarily. They had to be newly diagnosed with T2DM (WHO diagnostic criteria 1999), diagnosed for no more than 1 year, and have not received any previous medical treatment. Additionally, participants must have an HbA1c level of 7.5–10% at screening, as assessed by a local laboratory and at the pre-randomization visit, as assessed by a central laboratory, and a body mass index (BMI) between 19 and 40 kg/m² at screening.

Patients were excluded from the study if they met any of the following exclusion criteria: (1) congestive heart failure of New York Heart Association (NYHA) class III or IV or major cardiovascular events within 6 months before screening (significant cardiovascular history within the past 6 months prior to screening was defined as myocardial infarction, coronary angioplasty or bypass graft(s), valvular disease or repair, unstable angina pectoris, transient ischemic attack, or cerebrovascular accident). (2) Clinically apparent hepatobiliary disease, including but not limited to chronic active hepatitis and/or severe hepatic insufficiency. Alanine transaminase (ALT) or aspartate transaminase (AST), > 3x the upper limit of normal (ULN), or serum total bilirubin (TB) >34.2 μmol/L (2 mg/dL). (3) Estimated glomerular filtration rate (eGFR) < 45 mL/min per 1.73 m². (4) Diagnosis or history of acute metabolic diabetic complications, such as ketoacidosis and hyperglycemic hyperosmolar state, or diabetes insipidus within the past 6 months. (5) For women only, current pregnancy (confirmed by a positive pregnancy test) or breastfeeding. (6) Participation in any other study that included drug treatment in the past three months before enrollment. (7) Known hypersensitivity to dapagliflozin, metformin, or any of the excipients of the product. (8) Diagnosis or history of: a) chronic pancreatitis within the past 6 months or idiopathic acute pancreatitis within the past 4 weeks; b) gastrointestinal disease, for example, gastroenterostomy, enterectomy, Roemheld syndrome, severe hernia, intestinal obstruction, intestinal ulcer within the past 6 months; c) genetic galactose intolerance, LAPP lactase deficiency, and glucose-galactose malabsorption; d) organ transplantation or acquired immunodeficiency syndrome (AIDS) within the past 6 months; e) alcohol abuse or illegal drug abuse within the past 12 months; f) laser treatment for proliferative retinopathy within 6 months; g) stress condition, for example, surgery, serious trauma, etc., within the past 6 months, or planned surgery during the study period; h) chronic oxygen deficiency diseases, for example, pulmonary emphysema, pulmonary heart disease, within the past 6 months; i) type 1 diabetes mellitus (T1DM), diabetes resulting from pancreatic injury or secondary forms of diabetes, for example, acromegaly or (9) The subject being, in the judgment of the investigator, is unlikely to comply with the study protocol or has any severe concurrent medical or psychological conditions that may affect the interpretation of study results. (10) Involvement in planning and conducting the study.

This study protocol was approved by the ethical review board of Beijing Chaoyang Hospital of Capital Medical University with the certificate number of 2023-ke-772-3. This trial was registered at www.clinicaltrials.gov (NCT06327815). The trial protocol was in accordance with the SPIRIT guideline.

### 2.3 Endpoints

The primary endpoint of the study was the change in HbA1c level from baseline after 24 weeks of treatment. The secondary endpoints included the following: (1) The proportion of patients achieving HbA1c below 7.0%, absolute change in fasting plasma glucose/postprandial glucose (FPG/PPG) from baseline to week 24, and (2) the difference in satisfaction scores between the two groups measured using the diabetes treatment satisfaction questionnaire (DTSQ) at week 24. (3) Change from baseline in the diabetes quality of life (DQOL) questionnaire scores at weeks 12 and 24. (4) The differences in patient medication usage between the two groups were measured with adherence to refills and medications scale for diabetes (ARMS-D) at week 24. (5) Adverse events (AE), serious adverse events (SAE), and adverse drug reactions (ADRs). (6) Vital signs. (7) Physical examination. (8) Clinical laboratory indices. (9) Electrocardiogram

In the exploratory substudy, the primary exploratory endpoint was the proportion of time in the tight target range (TITR, 3.9-7.8 mmol/L). Secondary exploratory endpoints included time in range (TIR, i.e., the percentage of readings and time that a person spends with their blood glucose levels in a target range of 3.9–10 mmol/L), time below range (TBR, i.e., the percentage of readings and time that a person spends with their blood glucose levels below the target range), time above range (TAR, i.e., the percentage of readings and time that a person spends with their blood glucose levels above the target range), mean amplitude of glycemic excursions (MAGE, the mean blood glucose values exceeding one standard deviation (SD) from the 24‐hour mean blood glucose, which is used as an index of glycemic variability), and standard deviation of blood glucose (SDBG), which reflects the amount of variation or dispersion of a series of glucose values.

### 2.4 Randomization and study interventions

Patients were centrally randomized (1:1) to one of the two treatment groups using an Interactive Response Technology/Randomization and Trial Supply Management (IRT/RTSM). Prior to the study initiation, contact information and login directions for the system were provided to each site. Following randomization, patients in the FDC group received a single tablet containing 10 mg of dapagliflozin and 1000 mg of metformin hydrochloride XR once daily. Patients in the co-administration group received one tablet of 10 mg dapagliflozin once daily, and metformin hydrochloride XR, administered as two 500 mg tablets once daily with meals, or as one 500 mg tablet twice daily with meals if the patient is intolerant to the two-tablet dose. This study did not involve dose adjustments for study medications. The duration of treatment was 24 weeks.

The rescue treatment criteria from week 4 to week 12 were based on an FPG level exceeding 13.32 mmol/L, confirmed by a second measurement 3–5 days later. The rescue treatment criteria from weeks 12 to 24 were based on an FPG level exceeding 11.10 mmol/L (23). Rescue medications include but are not limited to metformin, GLP-1 receptor agonists, DPP-4 inhibitors, thiazolidinediones, sulfonylureas, and insulin. Patients administered rescue medications will continue to participate in the study and the results will be independently analyzed.

Subjects had to discontinue the use of prescription or over-the-counter medications (including vitamins, recreational drugs, dietary supplements, or herbal supplements) 7 days prior to the start of the study intervention (or 14 days in case the medication is a potential enzyme inducer) or 5 half-lives of the medication (whichever is longer) until the end of follow-up, unless the investigator and sponsor believe the medication will not interfere with the study. Acetaminophen/paracetamol with a dosage of ≤2 g/day was allowed at any time during the study period.

### 2.5 Data collection and management

As shown in **Table 1**, medical history and demographic information were collected during the screening phase. In addition, urine or blood pregnancy tests (for females only), physical examinations, vital signs, height, weight, waist circumference, 12-lead electrocardiogram (ECG), routine blood test, routine urine test, blood biochemistry, adverse events, HbA1c, fasting blood glucose, fasting insulin + C-peptide, postprandial 2h blood glucose, and postprandial 2h insulin + C-peptide levels will be assessed, and concomitant medications (antidiabetic and other drugs) will be recorded.

**Table 1:** Schedule of activities.

Study period	Screening period	Treatment period			
Visit day¹	V0	V1^2^	V2	V3 (end of treatment)	V4 (Follow-up)
Study week	≤2 weeks	Baseline	12w ± 5d	24w ± 5d	End of treatment+7d ± 3d
Sign informed consent forms^3^	X				
Eligibility (inclusion/exclusion criteria)	X	X			
Demographic	X				
Family history of diabetes	X				
History of diabetes and complications	X				
Medical/Current conditions	X			X	
Treatment					
Randomization		X			
Drug dispensation and accountability		X	X	X	
Safety assessments					
Pregnancy test (urine/blood, females only)	X	X	X	X	
Physical examination	X	X	X	X	
Vital signs (blood pressure, heart rate)	X	X	X	X	
Height	X				
Body weight	X	X	X	X	
Waist circumference		X	X	X	
12-lead electrocardiogram	X	X	X	X	
Hematology^4^	X	X	X	X	
Urinalysis^5^ (urine routine + urea albumin creatinine ratio)	X	X	X	X	
Biochemistry^6^	X	X	X	X	
Adverse events	X	X	X	X	X
Efficacy assessments					
Glycated hemoglobin ^7^	X	X	X	X	
Fasting plasma glucose (oral glucose tolerance test + C-peptide release test + insulin release test)	X	X	X	X	
2hr-plasma glucose^8^ (oral glucose tolerance test + C-peptide release test + insulin release test)	X	X	X	X	
Fasting insulin + c-peptide (oral glucose tolerance test + C-peptide release test + insulin release test)	X	X	X	X	
2hr-insulin, c-peptide^8^ (oral glucose tolerance test + C-peptide release test + insulin release test)	X	X	X	X	
Fasting plasma glucose (fingertip blood, in hospital)			X	X	
2h-plasma glucose (fingertip blood, in hospital)			X	X	
Continuous glucose monitoring index (time in tight target range\time in range\time above range\time below rang)			X		
Other assessments					
Concomitant glucose lowering medication collection	X	X	X	X	X
Concomitant other medication collection	X	X	X	X	X
Questionnaire					
Diabetes quality of life		X	X	X	
Diabetes treatment satisfaction questionnaire			X	X	
Adherence to refills and medication scale for diabetes			X	X	

^1^Visit Day: Visits will be conducted by phone at V4 for safety follow-up; patients should return to the hospital at V1 (baseline), V2 (12 weeks), and V3 (24 weeks).

^2^V1 (Baseline): If the date of blood test (including Hematology, Biochemistry, HbA1c, Fasting blood glucose, 2hr-plasma glucose, fasting insulin+c-peptide, 2hr-insulin+c-peptide) and Urinalysis at V0 is ≤ 2 weeks from V1, there is no need to be repeated at V1, and other tests except for that will still have to be performed at V1.

^3^Signing of ICF(s): Patients will be required to sign the ICF for the main study and the CGM sub-study.

^4^Hematological test:Absolute neutrophil count, Hemoglobin, Total white cell count, Absolute lymphocyte count, Platelet count

^5^Urinalysis: Urine routine, urine albumin-to-creatinine ratio (UACR).

^6^Biochemistry: Aspartate transaminase (AST), alanine transaminase (ALT), Albumin, Creatinine (Cr), Total Bilirubin, Blood urea nitrogen (BUN)/blood urea (urea), creatine phosphokinase (CPK), high-density lipoprotein cholesterol (HDL-C), low-density lipoprotein cholesterol (LDL-C), total cholesterol (TC), and triglycerides (TG).

^7^HbA1c will be tested in a local laboratory during the screening period. HbA1c will be tested in the central laboratory during the pre-randomization visit and treatment period. The HbA1c level will be tested by the central laboratory at V1, and the results will be obtained within 2 weeks. Eligible patients will be randomized according to the test results.

^8^2hr-plasma glucose/2hr-insulin, c-peptide: The time window for blood sampling is ±10min.

During the treatment period, patients will visit the hospital for follow-up at weeks 0, 12 (± 5 days), and 24 (± 5 days). The follow-up will include medication dispensing and management, urine or blood pregnancy test (for females only), physical examinations, vital signs, weight, waist circumference, 12-lead ECG, blood routine test, urine routine test, blood biochemistry, adverse events, HbA1c, fasting blood glucose, fasting insulin + C-peptide, postprandial 2h blood glucose, postprandial 2h insulin + C-peptide levels, and recording of concomitant medications (antidiabetic and other drugs). At weeks 12 (± 5 days) and 24 (± 5 days), fasting fingertip blood glucose and postprandial 2h fingertip blood glucose levels were measured. At week 12 (± 5 days), CGM was performed for approximately five days. One week (± 3 days) after the end of treatment, a telephonic follow-up will be conducted to record adverse events and concomitant medications (antidiabetic and other drugs).

Patient satisfaction, quality of life, and adherence were evaluated using DTSQ, DQOL questionnaire, and ARMS-D. The patients completed the DQOL questionnaire at week 0 and the DTSQ, DQOL questionnaire, and ARMS-D at weeks 12 and 24.

All patient data related to the study will be recorded on case report forms (CRFs), unless electronically transmitted to AstraZeneca or a designee (e.g., central laboratory). The investigator must maintain an accurate documentation (source data) to support the information entered in each CRF. The investigator must allow for monitoring, auditing, Institutional Review Board (IRB)/Independent Ethics Committee (IEC) review, and regulatory agency inspections related to the study, thereby providing direct access to source data files. Records and documents related to this study, including signed informed consent forms (ICFs), must be retained by the investigator for at least 25 years after study completion or as required by local regulations. Each subject was assigned a unique identifier to ensure privacy of the included participants. The data transmitted will not include the participant’s name or any personally identifiable information; only identifiers to distinguish the subjects will be used.

### 2.6 Sample size

The primary objective of this study was to demonstrate the non-inferiority of the FDC regimen of dapagliflozin/metformin XR to co-administered dual therapy of dapagliflozin and metformin for changes from baseline to week 24 in HbA1c within a non-inferiority margin of 0.30%, assuming a standard deviation of 1.2% (24). At a 1-sided significance level of 0.025, 253 patients per arm will be needed to provide 80% power (given a true difference of zero between the FDC regimen of dapagliflozin/metformin and XR and co-administered dual therapy of dapagliflozin and metformin). Assuming a dropout rate of 20%, 632 patients were randomized.

For the CGM sub-study, though exploratory in nature, the sample size was calculated based on exploratory hypothesis testing of the primary exploratory endpoint, which is the proportion of TITR (3.9-7.8 mmol/L) at week 12. Assuming a mean difference of 10%, standard deviation of 30%, and 2-sided significance level of 0.05, 143 patients per arm will provide 80% power for exploratory superiority testing. Assuming a dropout rate of 20%, approximately 358 patients were needed for the sub-study.

### 2.7 Statistical analysis

The full analysis set (FAS) included all randomized patients with documented baseline assessment and at least one post-baseline assessment for the primary efficacy endpoint. The per-protocol analysis set (PPS) will include patients without an important protocol deviation that might affect the primary efficacy analyses. The CGM analysis set will include patients from the full analysis set administered at least one dose of the study treatment prior to CGM performance, as well as CGM-evaluable data. The safety analysis set (SS) included all randomized patients who were administered at least one dose of the study treatment.

Primary efficacy analysis will be performed on the PPS, excluding HbA1c measurements after receiving rescue medications or permanent discontinuation of the study intervention. Missing data at week 24 will be handled using the multiple-imputation approach. Analysis of covariance (ANCOVA) will be used for the primary analysis. Point estimates and 2-sided 95% confidence intervals for the mean change within each treatment group, as well as the difference in mean change between the two groups, will be provided. The dapagliflozin/metformin FDC regimen was considered non-inferior to dapagliflozin and metformin co-administered if the upper limit of the 2-sided 95% (or 1-sided 97.5%) confidence interval of the difference in mean change in HbA1c from baseline to week 24 between the groups was below 0.3%.

The secondary endpoints will be analyzed in the PPS. An ANCOVA model will be used to analyze the absolute changes in FPG and PPG from baseline to week 24, reporting the point estimates of the average changes and two-sided 95% confidence intervals (CIs) within each treatment group as well as the differences in mean changes between groups. The difference in the proportion of patients achieving HbA1c levels below 7.0% at week 24 will be analyzed using logistic regression, adjusting for baseline HbA1c values. Participants with missing HbA1c measurements at week 24 will have the values imputed by dichotomizing the imputed HbA1c values at week 24. Odds ratios (ORs) and 95% confidence intervals will be reported. Descriptive statistics will be used for DQOL, DTSQ, and ARMS-D scores. An ANCOVA model will analyze the changes in the DQOL score from baseline to weeks 12 and 24. Point estimates of mean changes and 95% CIs within each group were calculated along with the differences in mean changes between the two groups. Differences in DTSQ and ARMS-D scores at week 24 between the two groups will be compared using the t-test or Wilcoxon rank-sum test, based on the normality and homogeneity of variances of the data.

The analyses for the primary and secondary endpoints will be repeated using FAS to examine the robustness of the results.

Exploratory endpoints will be analyzed based on the CGM analysis set. Point estimates and 95% CIs for all exploratory outcomes within each group were calculated along with estimates and 95% CIs for the differences between the two groups.

Safety analysis will be based on SS, summarizing the numbers and incidence rates of AEs, SAEs, and ADRs in each treatment group. At each scheduled time point, descriptive statistics will be used to summarize the values and changes from the baseline in vital signs, physical examinations, and clinical laboratory parameters within the different groups.

## 3 Discussion

T2DM is a significant global health issue, and poor glycemic control can lead to severe complications including chronic kidney disease, retinopathy, neuropathy, stroke, and myocardial infarction (25, 26). Therefore, controlling blood glucose levels and delaying the onset of complications is crucial for diabetes treatment. Recent clinical guidelines suggest that metformin and SGLT2 inhibitors can be used as initial dual therapy in patients with high HbA1c levels (2).

To date, studies comparing FDC to coadministration are rare. XIGDUO^®^ XR was launched in China in June 2023, and head-to-head clinical trials comparing the initial combination treatment with metformin and SGLT2 inhibitors with FDC therapy are still lacking. This study is a multicenter, non-inferiority, open-label randomized controlled trial designed to compare dapagliflozin/metformin XR FDC with co-administration of dapagliflozin and metformin XR in terms of glycemic control, patient satisfaction, quality of life, adherence to treatment, and safety in patients with newly diagnosed type 2 diabetes mellitus (T2DM). The primary endpoint focused on glycemic control efficacy, specifically the change in HbA1c from baseline after 24 weeks of treatment. Patient satisfaction, quality of life, and adherence to treatment were measured using the DTSQ, DQOL, and ARMS-D, respectively. The DTSQ has proven valuable for understanding and measuring patients’ treatment satisfaction in assessments of new treatments and strategies (27). DQOL, specifically developed to evaluate the quality of life of individuals with diabetes, is widely used and helps to understand how diabetes and its treatment impact the overall well-being and day-to-day experiences of patients (28, 29). The ARMS-D is a reliable and valid questionnaire designed to assess medication adherence in individuals with diabetes and helps healthcare providers identify adherence barriers and tailor interventions to improve compliance and health outcomes (30).

The multicenter design of this study enhances the generalizability and representativeness of the results. The change in HbA1c was used as the primary endpoint, as HbA1c reflects the patient’s blood glucose levels over the past 3–4 months and is considered the gold standard for blood glucose control. Therefore, the study endpoint reflects the effect of glycemic control. Additionally, CGM was employed as an exploratory study to assess glucose variability in more detail, offering deeper insights into future treatment strategies.

This study had several limitations. First, the open-label design may introduce a potential positive bias toward the FDC group, particularly concerning the DTSQ and ARMS-D scores, given the known convenience of a single tablet regimen. Second, the 24-week duration of the trial limited our ability to assess long-term outcomes, including the trajectory of β-cell function, sustainability of glycemic control, and more comprehensive safety profile. Third, the recruitment of participants exclusively from Chinese centers may limit the generalizability of our findings to other ethnic groups and healthcare settings. While this focus provides highly relevant data for the Chinese population, future multinational studies are warranted to confirm the global applicability of the results.

In conclusion, as the first study to directly compare the dapagliflozin/metformin XR FDC with the separate administration of dapagliflozin and metformin hydrochloride XR, this scientifically designed trial has the potential to provide crucial evidence supporting the use of FDCs. Additionally, it may offer valuable insights for physicians in selecting treatment regimens for diabetes and in improving patient adherence.
